# Printed Zinc Paper Batteries

**DOI:** 10.1002/advs.202103894

**Published:** 2021-11-05

**Authors:** Peihua Yang, Jia Li, Seok Woo Lee, Hong Jin Fan

**Affiliations:** ^1^ School of Physical and Mathematical Sciences Nanyang Technological University Singapore 637371 Singapore; ^2^ Rolls‐Royce@NTU Corporate Lab Nanyang Technological University Singapore 639798 Singapore; ^3^ School of Electrical and Electronic Engineering Nanyang Technological University Singapore 639798 Singapore; ^4^ Innovative Centre for Flexible Devices (iFLEX) Nanyang Technological University Singapore 639798 Singapore

**Keywords:** hydrogel, paper electronics, printed battery, self‐powered system, zinc batteries

## Abstract

Paper electronics offer an environmentally sustainable option for flexible and wearable systems and perfectly fit the available printing technologies for high manufacturing efficiency. As the heart of energy‐consuming devices, paper‐based batteries are required to be compatible with printing processes with high fidelity. Herein, hydrogel reinforced cellulose paper (HCP) is designed to serve as the separator and solid electrolyte for paper batteries. The HCP can sustain higher strain than pristine papers and are biodegradable in natural environment within four weeks. Zinc‐metal (Ni and Mn) batteries printed on the HCP present remarkable volumetric energy density of ≈26 mWh cm^–3^, and also demonstrate the feature of cuttability and compatibility with flexible circuits and devices. As a result, self‐powered electronic system could be constructed by integrating printed paper batteries with solar cells and light‐emitting diodes. The result highlights the feasibility of hydrogel reinforced paper for ubiquitous flexible and eco‐friendly electronics.

## Graphical abstract

Hydrogel reinforced cellulose paper presents enhanced mechanical and conductivity properties that make them suitable as the separator and quasi‐solid electrolyte for paper batteries. The printed zinc batteries (Mn–Zn and Ni–Zn) are flexible, cuttable, and potentially integrable with other paper electronics.

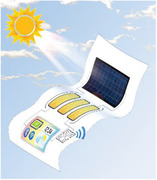

## 1 Introduction

As fifth generation (5G) broadband has been set for commercial availability since 2020, full potential of Internet of Things (IoT) technology will be released by allowing the interconnection and communication of massive amount of IoT devices.^[^ ^1^, ^2^ ^]^ The ever‐growing demand of IoT devices in smart utilities and healthcare applications spurs innovation and development of wearable and flexible electronics.^[^ ^3^, ^4^, ^5^, ^6^ ^]^ Burgeoning development of flexible electronics not only poses severe challenges to traditional manufacturing methods,^[^ ^7^ ^]^ but also raises further concerns on sustainability, in terms of the waste disposal and recyclability at the end of product life.^[^ ^8^, ^9^ ^]^ Cellulose paper, derived from abundant natural biomass on earth,^[^ ^10^, ^11^ ^]^ presents an ideal building block for developing flexible and environmentally sustainable electronics. Because of its portability, renewability and function tuneability, paper electronics could be the raising star of next‐generation functional devices,^[^ ^12^ ^]^ especially in the field of paper‐based flexible batteries,^[^ ^13^ ^]^ sensors,^[^ ^14^ ^]^ circuits,^[^ ^15^ ^]^ and diagnostic equipment.^[^ ^16^ ^]^ Imagine if these devices can be integrated on a piece of paper, it will greatly facilitate the application scenarios and enhance the commercial values. Meanwhile, the compatibility of paper to printing techniques allows facile ink printing of a wide variety of source materials,^[^ ^17^, ^18^ ^]^ further flourishing the paper electronics.

Among these printed paper devices, batteries are considered as the heart of paper electronics.^[^ ^19^ ^]^ Compared with conventional lithium‐ion batteries, aqueous zinc‐ion batteries hold the advantages of low cost, high theoretical volumetric capacity (5854 Ah L^–1^), good rechargeability, and safe chemistry.^[^ ^20^, ^21^, ^22^, ^23^, ^24^ ^]^ Paper‐based zinc‐ion batteries are mainly shown in two categories. One is assembling active materials on the surface of cellulose fibers paper or directly fabricating freestanding active material films as paper‐like electrodes.^[^ ^25^, ^26^, ^27^ ^]^ This method hinders the printability of paper, making it almost impossible to use printing technology to fabricate batteries. The other type of paper zinc‐ion batteries employs the paper only as the substrate, in which the batteries are in‐plane printed on one side and present interdigitated configuration.^[^ ^28^, ^29^ ^]^ Printing electrode inks on both sides is challenging because it may cause short of the battery due to the rough and permeable surface of paper. Accordingly, the printed micro‐batteries on paper deliver limited areal capacity and volumetric energy density (up to ≈10 mWh cm^–3^). The above limitations of energy storage components impede the integration and reliability of paper electronics.

Herein, we propose a new hydrogel reinforced cellulose paper (HCP) for printable zinc batteries and paper electronics. By combining the characteristics of cellulose paper and hydrogel, the composite paper presents favorable mechanical and conductivity properties that are suitable to its function as the separator and electrolyte for quasi‐solid zinc batteries. The anode and cathode materials are printed on the front and back of the paper, respectively. This is different from previous nano‐cellulose/hydrogel composites where the composites were employed only as the separator for energy devices,^[^ ^30^, ^31^ ^]^ but the electrode materials cannot be printed on the composite. In addition, the compatibility of the paper batteries with flexible circuits and devices allows the construction of a self‐powered paper system that integrates printed battery with circuit lines and solar cells. This work demonstrates the possibility of printed paper batteries and integration with flexible electronics toward the new era of paper electronics.

## 2 Results and Discussion

**Figure 1:**
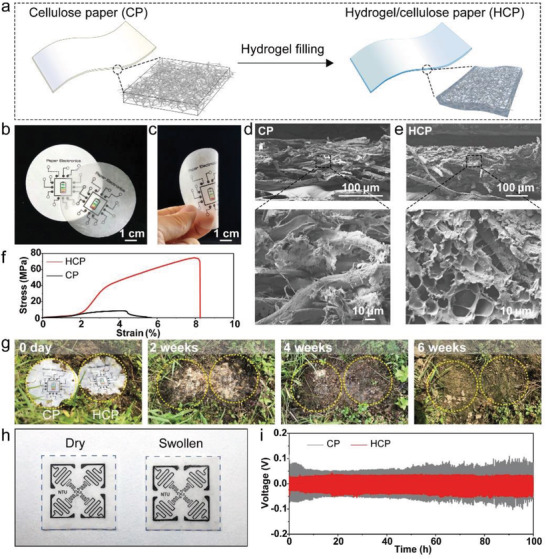
Fabrication and characterizations of hydrogel cellulose paper (HCP). a) Schematic of original CP and HCP. b) Optical image of HCP (top) and original CP (bottom) with printed circuit patterns. c) One piece of HCP under mechanical deformation. SEM images of d) CP and e) HCP. f) Stress–strain curves of HCP and CP. g) The biodegradability tests of CP (left) and HCP (right) under moist soil. Yellow dash circles show outline of the samples. h) Photograph of a dry HCP (left) and a swollen HCP (right) with printed patterns. Blue dash squares with the same size of 30 mm × 30 mm show the boundaries of HCP. i) Galvanostatic plating/stripping curves of zinc symmetrical cells at a current density of 5 mA cm^−2^ and a cut‐off capacity of 1 mAh cm^−2^.

To fabricate the HCP, commercial cellulose filter paper (CP) was pre‐immersed in acrylamide solution with initiator and crosslinking agent. After heating under inert atmosphere, polyacrylamide (PAAM) hydrogel was polymerized among the fiber gaps in CP (**Figure** **1**a). Since the hydrogel completely fills the paper, light scattering through HCP is greatly reduced, thus the fabricated HCP becomes semi‐transparent (Figure 1b). Patterns and circuits can be printed directly on the HCP, as well as on the original CP. In addition, the fabricated HCP is flexible and can tolerate mechanical deformations (Figure 1c). Hence, it indicates that the HCP maintains the intrinsic printability and flexibility of paper, which ensures its potential application in paper/printing industry.

Scanning electron microscopy (SEM) images in Figure 1d,e show morphology of paper before and after polymerization. The original CP is mainly composed of cellulose fibers with diameters of 10–20 µm. After polymerization, PAAM fills in gaps between cellulose fibers of CP and thus increases its thickness from 100 µm to ≈230 µm. In the freeze‐dried HCP, hydrogel with micro pores surrounds the cellulose fibers. The hydrogel has an evident reinforcement to the mechanical properties of composite paper (Figure 1f). When the strain is less than 2%, the HCP has no difference from the original CP. Their large strain in spite of low stress is caused by angular translation of randomly oriented fibers to be aligned to the direction of applied stress. At over 2% strain, the HCP exhibits a larger Young's modulus (about 3.0 GPa) than the CP because hydrogel in the HCP prevents slip of fibers and their axial strain is fully responsible to the stress while the CP has severe slip of fibers and consequent stretching. Eventually, the HCP reaches a tensile strength of 75 MPa, which is nine times that of the original CP (8.3 MPa). High strength of the HCP endows paper batteries be more adaptive under various mechanical deformation conditions.

The HCP holds great biodegradability as natural cellulose. When buried in natural soil Singapore (roof garden, NTU campus), both CP and HCP became fractured after two weeks of burial (Figure 1g). PAAM and cellulose are decomposable in the presence of bacteria, fungi, and other microorganisms.^[^ ^32^, ^33^ ^]^ After four weeks, HCP are completely degraded while the CP takes six weeks for full degradation. Hygroscopicity of hydrogel greatly facilitates the growth and reproduction of microorganism. For comparison, Celgard separator (a commonly used separator in battery industrial composed of polypropylene/polyethylene^[^ ^34^ ^]^) and other three typical polymer separators (cellulose acetate (CA), polyethersulfone (PES) and polytetrafluoroethylene (PTFE)) retained their original shape after the same burial time (Figure S1, Supporting Information). Therefore, HCP with high mechanical strength and good biodegradability holds great promise for next‐generation sustainable paper electronics.

It is worth noting that the HCP only expands itself in longitudinal direction (i.e., increases thickness), and presents neglectable expansion in the lateral direction when it is immersed and swollen in electrolyte solution. This can be attributed to the anisotropic expansion of hydrogel which is constrained in the laminate stacking frame structure of cellulose fibers. This property guarantees the integrity of the printed electrode films during the activation of HCP electrolyte. As a proof of concept, radio‐frequency identification (RFID) antenna patterns were printed with a commercial printer on two pieces of HCP with the same size of 30 mm × 30 mm. One patterned HCP was swollen in liquid electrolyte. As shown in Figure 1h, the lateral size of the swollen HCP had negligible change compared with the dry one, despite the obvious increase in thickness (Figure S2, Supporting Information). Thus, the printed pattern maintains its integrity and conformability on the swollen HCP.

To check the performance of HCP as an electrolyte layer, it was soaked in an alkaline KOH/LiOH solution. The completely swollen HCP delivers a conductivity of 33 mS cm^−1^ from the impedance measurement (Figure S3, Supporting Information). The value is a little lower than that of pure alkaline hydrogel electrolyte (≈77–100 mS cm^−1^),^[^ ^35^, ^36^, ^37^ ^]^ which can be rooted to the lower content of hydrogel in HCP. Compared with aqueous electrolyte in pure cellulose paper, the HCP electrolyte shows highly stable Zn plating/stripping behavior and smaller polarization (Figure 1i). This enhancement is ascribed to the hydrogel which can suppress zinc dendrites formation (Figure S4, Supporting Information), and thus enhance the batteries cycling stability.^[^ ^38^ ^]^

**Figure 2:**
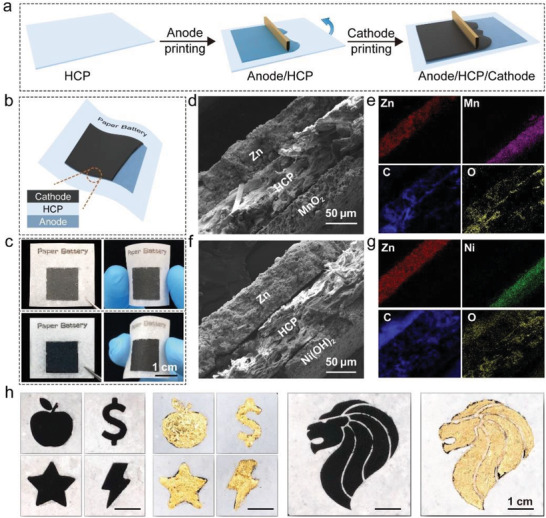
Structures of printed zinc batteries on HCP. a) Schematic of the printing process. b) Illustration of a printed battery. c) Optical images of a printed Mn–Zn battery when the HCP layer is dry (top) and swollen (bottom). d) Cross‐sectional SEM image of Mn–Zn battery and e) corresponding EDS element mappings. f) Cross‐sectional SEM image of Ni–Zn battery and g) corresponding EDS element mappings. h) Various patterned paper batteries with and without gold foil current collectors.

To demonstrate a paper battery using the HCP, we printed battery electrodes on front and back of the HCP using screen printing (**Figure** **2**a). Herein, manganese‐zinc (Mn–Zn) and nickel‐zinc (Ni–Zn) are printed for examples, but it is applicable to other metals. The successful printing of battery relies on the customized formulation of electrode inks. The Ni(OH)_2_ and MnO_2_ were synthesized by a facile co‐precipitation method (Figure S5, Supporting Information). The anode ink was composed of Zn microparticles, ZnO, carbon black and polyvinylidene difluoride (PVDF). The cathode ink was mainly composed of Ni(OH)_2_ (or MnO_2_) powders with a certain amount of carbon black and PVDF. The carbon black enhances the electronic conductivity of the printed electrode, and the PVDF additive improves the bonding of electrodes and HCP substrate. The cathode and anode inks are printed on the front and back of HCP, respectively (Figure 2b). The printed battery is flexible and durable under mechanical deformation both in dry condition and after hydrogel swelling (Figure 2c).

The microstructure of the printed batteries is revealed by cross‐sectional SEM and corresponding EDS elements mapping images (Figure 2d–g). The hydrogel filled in HCP provides a dense separator which effectively prevents the mixing of anode and cathode inks, and thus avoids short‐circuit (Figure S6, Supporting Information). The thickness of a dry printed device is about 200 µm. After swelling by electrolyte absorption, the thickness increases to ≈400 µm. The particles of zinc, Ni(OH)_2_ and MnO_2_ are surrounded by activated carbon, with sizes of tens nanometers (Figure S7, Supporting Information). After the HCP is completely soaked with liquid electrolyte, gold foil current collectors are attached on the anode and cathode. The gold foil is used herein for the evaluation of battery performance. Finally, we demonstrate the paper batteries can be printed in various shapes and complex geometries (Figure 2h), which makes the screen printing a low‐cost and high‐throughput film technique for flexible zinc batteries.

The liquid electrolytes absorbed by the HCP present a low vapor pressure at room temperature (1.8 kPa at ≈25°C, Figure S8, Supporting Information), which is quite close to the partial pressure of water vapor in ambient lab environment with the relative humidity of 60% (1.9 kPa, 25°C). Therefore, the wet HCP can maintain moisture equilibrium with the ambient environment and keep stable even exposed to the air.^[^ ^38^ ^]^ After swollen in the liquid electrolytes, the printed batteries can keep their weight for over one week in ambient environment (Figure S9, Supporting Information). Consequently, such HCP based battery can work without extra packaging, enabling its higher‐level of integration with other electronic components on paper‐based electronics.

**Figure 3:**
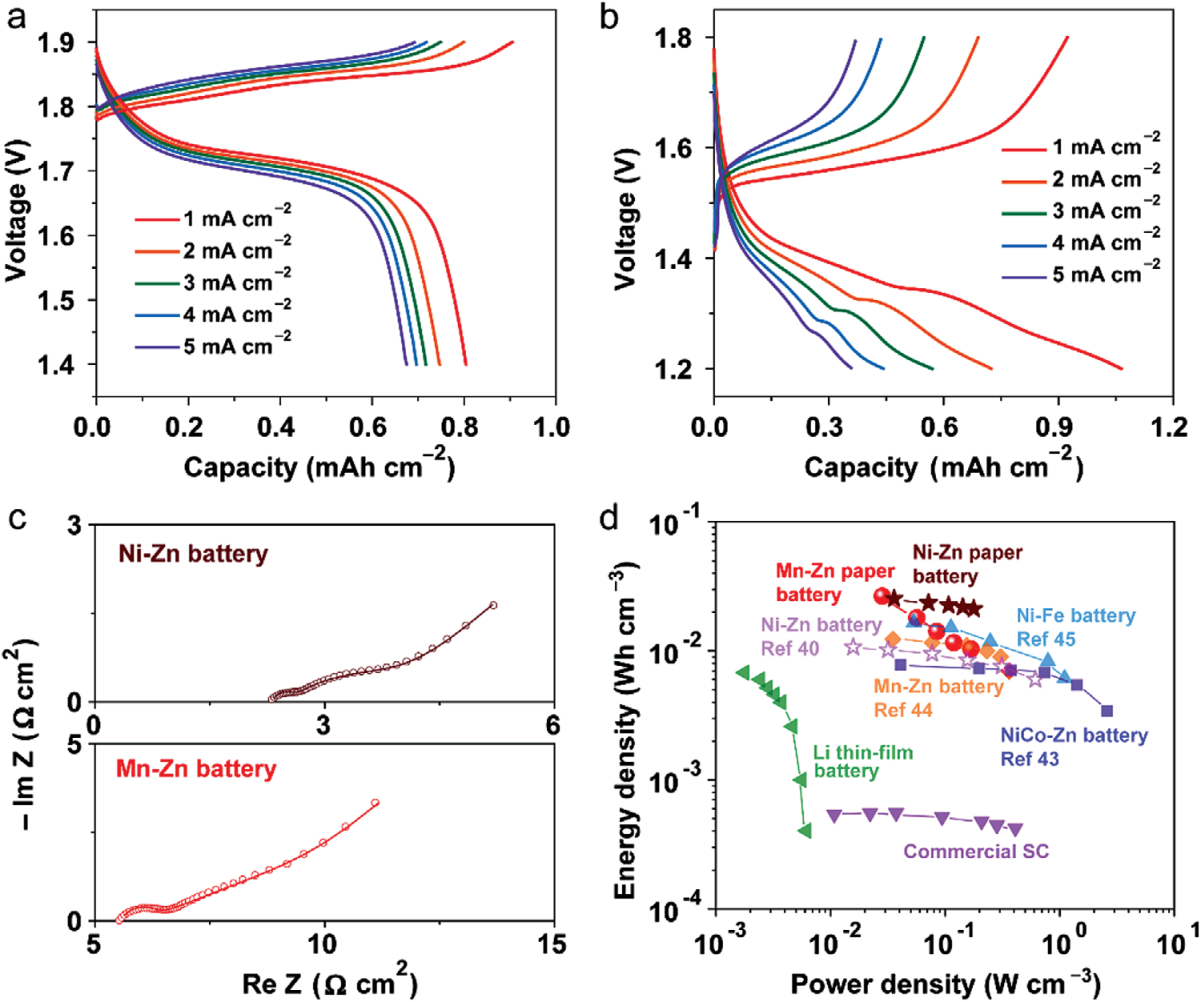
Electrochemical performance of printed batteries. The charge and discharge curves of a) Ni–Zn and b) Mn–Zn batteries at various current densities. c) Impedance profiles of printed batteries. d) Ragone plot of printed paper batteries compared with other energy storage devices (where the volume refers to that of a whole cell).

The electrochemical and mechanical properties of the printed Ni–Zn and Mn–Zn batteries are thoroughly characterized. For flexible and thin devices, it has been pointed out that the areal and volumetric performance metrics are crucial.^[^ ^39^ ^]^ From the charge and discharge curves (**Figure** **3**a,b), the areal capacity of Ni–Zn and Mn–Zn batteries is determined to be 0.8 and 1.1 mAh cm^−2^ (current density of 1 mA cm^−2^) according to discharge curves, respectively, which is equal to volumetric capacity of 20 and 27.5 mAh cm^−3^ (including the entire device volume). These values are significantly higher than previous flexible Ni–Zn batteries (4.4 mAh cm^−3^)^[^ ^40^ ^]^ and Mn–Zn batteries (3.3 mAh cm^−3^)^[^ ^41^ ^]^ under similar measured current densities. The plateaus in the charge and discharge curves correspond to the peaks in the cyclic voltammetry curves (Figure S10, Supporting Information). In Mn–Zn battery, the two plateaus at 1.25 and 1.35 V are attributed to zinc ion and proton intercalation in the cathode, respectively.^[^ ^42^ ^]^ In Ni–Zn battery, a reduction plateau at 1.7 V and an oxidation plateau at 1.85 V are observed, which can be ascribed to an overall electrochemical reaction: Zn + 2NiOOH+ H_2_O ↔ ZnO + 2Ni(OH)_2_. When the current density is five times higher (from 1 to 5 mA cm^−2^), the capacity of Ni–Zn battery slightly drops to ≈0.7 mAh cm^−2^, retaining 84% of the initial capacity. This rate capability is much better than that of Mn–Zn batteries (34% capacity retention when current density increases from 1 to 5 mA cm^−2^). The resistance of the two battery systems were also investigated using impedance spectra (Figure 3c). The equivalent internal resistance (including electrode resistance, electrolyte resistance and contact resistance) is 5.6 Ω cm^2^ in the Mn–Zn battery and 2.3 Ω cm^2^ in the Ni–Zn battery. The high resistance in the Mn–Zn battery originates from the relatively low conductivity of HCP swollen in ZnCl_2_ mixture electrolyte (18 mS cm^−1^, Figure S11, Supporting Information), which is only half of the conductivity of HCP soaked in KOH/LiOH mixed electrolyte.

The volumetric energy and power densities of printed paper batteries are evaluated and compared to other advanced energy storage devices in recent literature (Figure 3d). Due to the package‐free device configuration and high mass loading of active materials on HCP, our Ni–Zn paper battery achieves a maximum volumetric energy density of 26.6 mWh cm^–3^ with a power density of 25.0 mW cm^–3^. Similarly, the Mn–Zn paper battery presents a maximum volumetric energy density of 25.4 mWh cm^–3^ with a power density of 31.6 mW cm^–3^. These values are more than twice that of the flexible Ni–Zn batteries (10.7 mWh cm^−3^ based on total device volume).^[^ ^40^ ^]^ They are also much higher than the reported flexible NiCo‐Zn battery (8.0 mWh cm^−3^),^[^ ^43^ ^]^ Mn–Zn battery (12.0 mWh cm^−3^),^[^ ^44^ ^]^ and Ni–Fe battery (16.6 mWh cm^−3^).^[^ ^45^ ^]^ By evaluating the volumetric (total device) energy and power densities, our printed paper batteries may represent the state‐of‐the‐art and supersede the commercial supercapacitors and lithium‐ion thin‐film batteries (see Table S1, Supporting Information).^[^ ^46^, ^47^, ^48^, ^49^, ^50^, ^51^, ^52^, ^53^, ^54^, ^55^ ^]^

The printed batteries with HCP electrolyte exhibit good cycle performance with Coulomb efficiency over 95% (Figure S12, Supporting Information). After 500 charge/discharge cycles at a current density of 2 mA cm^−2^, the capacity of Ni–Zn and Mn–Zn batteries retained at ≈0.4 and 0.5 mAh cm^−2^, respectively. To prove the universality of the hydrogel reinforcement strategy, we also used commercial A4 copy paper for the fabrication of printed batteries (Figure S13, Supporting Information). The results have no significant difference compared with the printed batteries using cellulose filter paper.

**Figure 4:**
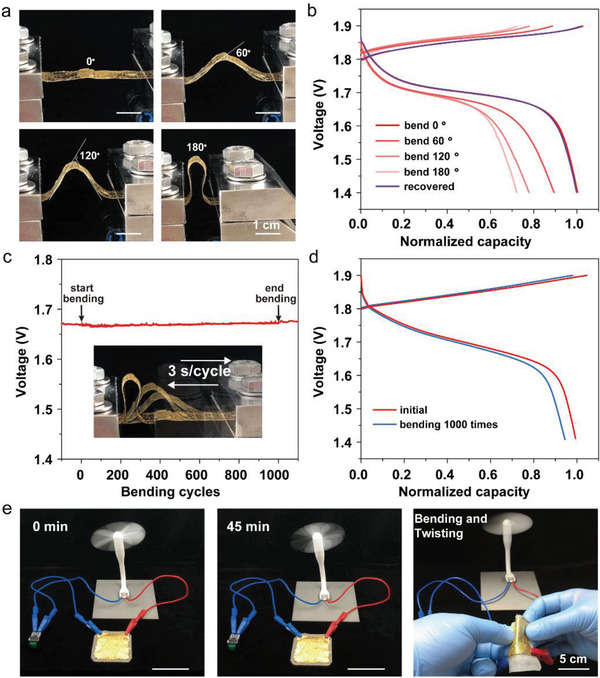
Performance of printed Ni–Zn batteries under mechanical deformations. a) Optical images of a 1 cm × 4 cm battery under various bending conditions and b) the corresponding charge and discharge curves. c) The voltage profile of battery while undergoing 1000 bending cycles. Inset illustrates the battery under repeated 180° bending cycles at a speed of 3 s per cycle. d) Assessing the bending cycles to the capacity of battery. e) Durability and mechanical deformation test of a 4 cm × 4 cm battery powering a mini electric fan (see also Video S1, Supporting Information).

Compared with coin or cylindrical cells, printed paper batteries have the unique advantages of mechanical flexibility. They can be bent and twisted without causing sharp performance failure. For this purpose, a 1 cm × 4 cm Ni–Zn battery was fabricated and cycled under various bending conditions (**Figures** **4**a,b). It can be found that the capacity of the 180°‐bent battery decreased to 72% of the flat condition. After the battery was restored to flat condition, the battery capacity is almost recovered to the original status. Furthermore, when the battery was subjected to 1000 cycles of bending, the open‐circuit voltage (after full charging) presents negligible fluctuation (Figure 4c). And only 5% decrease in capacity was observed after 1000 cycles of bending (Figure 4d). The small capacity loss may be caused by two reasons: the contact issue between current collector and electrodes, and cracks caused by excessive bending (indicated in Figure S14, Supporting Information), which are common in thin‐film energy storage devices. For a demonstration, a 4 cm × 4 cm battery was employed to power an electric fan for over 45 min continuously (Figure 4e). Bending and twisting the battery cause no major impact (see Video S1, Supporting Information), implying the robustness of the battery structure.

**Figure 5:**
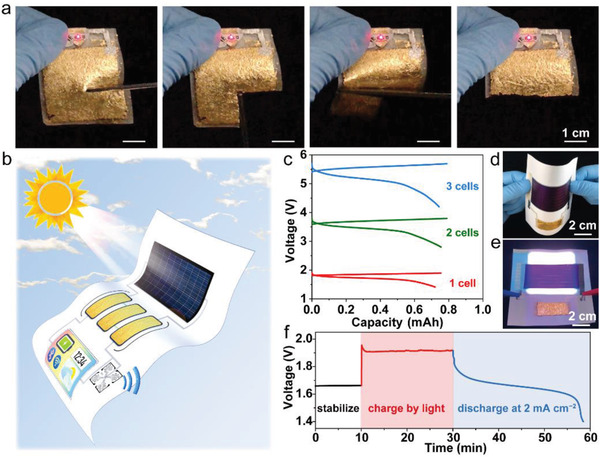
Demonstration of self‐powered paper electronics. a) Cuttability demonstration of the printed battery. b) Illustration of a self‐powered system on paper. c) Charge and discharge profiles of the printed Ni–Zn battery (1 cm × 1 cm) connected in series from one to three cells at a fixed current of 5 mA. d) Flexibility demonstration of a self‐powered system constructed by flexible solar cell, printed battery, and circuits. e) The paper system working under simulated sunlight. f) The voltage profile of paper battery with light charging and dark discharging.

For flexible energy devices composed of hydrogel electrolyte, safety, cuttability, and editability are their intrinsic features.^[^ ^56^, ^57^, ^58^ ^]^ To prove this, a 4 cm × 4 cm battery (fully charged) was employed and silver wires were printed on the same paper substrate to connect the electrodes to a red light‐emitting diode (LED). When the switch was turned on, the LED can emit light normally. As shown in **Figure** **5**a and Video S2 (Supporting Information), when the battery was cut off piece by piece, the luminous performance of LED was not disturbed, indicating that the cutting does not affect the functionality of the remaining battery nor causes short. This editability combined with mechanical flexibility may provide new opportunities in designing power units that are compatible with sophisticated flexible devices, for example, biomorphic robots.^[^ ^59^ ^]^

The best destination for the paper batteries should be in paper electronics. The integration of our battery and other flexible electronics on paper will promote the electronic system into a compact, lightweight, and convenient configuration. In principle, it is possible to achieve the all‐in‐one paper electronic system by integrating built‐in flexible solar cells, batteries, circuitry, and energy‐consuming electronics. This concept is schematically illustrated in Figure 5b. To test in this study, we first fabricated multiple Ni–Zn batteries connected in series to achieve high output voltage, which increases linearly from 1.7 V for a single unit to 5.1 V for three batteries (Figure 5c). A proof of this concept is demonstrated by a self‐powered paper energy system (Figure 5d). Instead of developing a printed paper solar cell which is beyond this study, we used a commercial thin‐film amorphous silicon solar cell (7 cm × 3 cm, *J*–*V* curve is shown in Figure S15, Supporting Information). A printed Ni–Zn battery (4 cm × 2 cm) was connected to the solar cell by silver circuits printed on the same paper. The entire paper system maintains flexibility of all components and can be bent according to operation requirements (Figure 5d).

To confirm the compatibility of the battery and solar cell on paper, the light charging performance of the integrated system was investigated. The battery was fully discharged first and stabilized for 10 min. Then the solar cell was illuminated by a standard solar simulator at one sun intensity (Figure 5e), and the solar cell started to charge the battery under ≈1.9 V for 20 min (Figure 5f). When the light was turned off, the battery was discharged at a constant current density of 2 mA cm^−2^. The total energy released from the battery is ≈7.5 mAh (i.e., ≈0.9 mAh cm^−2^), which is converted from light by the solar cell. Although we employed commercial solar cell in the experiment for conceptual demonstration, we are optimistic that with the fast advance in manufacturing technologies, more and more energy harvesting devices (such as solar cells^[^ ^60^, ^61^ ^]^ and nanogenerators^[^ ^62^ ^]^) can be printed on paper. Therefore, our printed zinc‐metal batteries may serve as reliable and low‐cost energy storage units in an all‐paper self‐powered system.

## 3 Conclusion

In summary, we have realized zinc‐based paper batteries by printing electrode inks on biodegradable hydrogel reinforced cellulose paper. The hydrogel paper serves as both electrolyte and separator for printed batteries, for which the hydrogel significantly improves the mechanical strength of the cellulose fibers and maintains the ion conductivity of the composite. As a result, the printed batteries are cuttable and flexible without the risk of short of contact failure. In addition, they exhibit high volumetric capacity and energy density which outperform other paper based or thin‐film energy storage units. The facile synthesis and high performance may push forward the advance of paper batteries for applications in printed electronics and integrated paper circuits.

## Conflict of Interest

The authors declare no conflict of interest.

## Supporting information

Supporting Information

Supplemental Video 1

Supplemental Video 2

## Data Availability

Research data are not shared.

## References

[1] M. Médard , Nat. Electron. 2020, 3, 2.

[2] I. Mistry , S. Tanwar , S. Tyagi , N. Kumar , Mech. Syst. Signal Process. 2020, 135, 106382.

[3] S. Park , S. W. Heo , W. Lee , D. Inoue , Z. Jiang , K. Yu , H. Jinno , D. Hashizume , M. Sekino , T. Yokota , K. Fukuda , K. Tajima , T. Someya , Nature 2018, 561, 516. 30258137 10.1038/s41586-018-0536-x

[4] J. Yun , Y. Zeng , M. Kim , C. Gao , Y. Kim , L. Lu , T. T.‐H. Kim , W. Zhao , T.‐H. Bae , S. W. Lee , Nano Lett. 2021, 21, 1659. 33533624 10.1021/acs.nanolett.0c04362

[5] M. Kim , I. D. Jung , Y. Kim , J. Yun , C. Gao , H.‐W. Lee , S. W. Lee , Sens. Actuators, B 2020, 322, 128601.

[6] L. Zhang , D. Chao , P. Yang , L. Weber , J. Li , T. Kraus , H. J. Fan , Adv. Energy Mater. 2020, 10, 2000142.

[7] C. Wang , K. Xia , H. Wang , X. Liang , Z. Yin , Y. Zhang , Adv. Mater. 2019, 31, 1801072. 10.1002/adma.201801072 30300444

[8] Y. H. Jung , T.‐H. Chang , H. Zhang , C. Yao , Q. Zheng , V. W. Yang , H. Mi , M. Kim , S. J. Cho , D.‐W. Park , H. Jiang , J. Lee , Y. Qiu , W. Zhou , Z. Cai , S. Gong , Z. Ma , Nat. Commun. 2015, 6, 7170. 26006731 10.1038/ncomms8170 PMC4455139

[9] L. Teng , S. Ye , S. Handschuh‐Wang , X. Zhou , T. Gan , X. Zhou , Adv. Funct. Mater. 2019, 29, 1808739.

[10] S. Wang , A. Lu , L. Zhang , Prog. Polym. Sci. 2016, 53, 169.

[11] F. Jiang , T. Li , Y. Li , Y. Zhang , A. Gong , J. Dai , E. Hitz , W. Luo , L. Hu , Adv. Mater. 2018, 30, 1703453. 10.1002/adma.201703453 29205546

[12] Z. Wang , Y.‐H. Lee , S.‐W. Kim , J.‐Y. Seo , S.‐Y. Lee , L. Nyholm , Adv. Mater. 2021, 33, 2000892. 10.1002/adma.202000892 32557867

[13] B. Yao , J. Zhang , T. Kou , Y. Song , T. Liu , Y. Li , Adv. Sci. 2017, 4, 1700107. 10.1002/advs.201700107 PMC5515121 28725532

[14] S. Cinti , D. Moscone , F. Arduini , Nat. Protoc. 2019, 14, 2437. 31270508 10.1038/s41596-019-0186-y

[15] S. Conti , L. Pimpolari , G. Calabrese , R. Worsley , S. Majee , D. K. Polyushkin , M. Paur , S. Pace , D. H. Keum , F. Fabbri , G. Iannaccone , M. Macucci , C. Coletti , T. Mueller , C. Casiraghi , G. Fiori , Nat. Commun. 2020, 11, 3566. 32678084 10.1038/s41467-020-17297-z PMC7367304

[16] M. M. Gong , D. Sinton , Chem. Rev. 2017, 117, 8447. 28627178 10.1021/acs.chemrev.7b00024

[17] Y. Zhang , L. Zhang , K. Cui , S. Ge , X. Cheng , M. Yan , J. Yu , H. Liu , Adv. Mater. 2018, 30, 1801588. 10.1002/adma.201801588 30066444

[18] P. Yang , H. J. Fan , Adv. Mater. Technol. 2020, 5, 2000217.

[19] S. Lanceros‐Méndez , C. M. Costa , Printed Batteries: Materials, Technologies and Applications, John Wiley & Sons Ltd, New York 2018.

[20] J. F. Parker , C. N. Chervin , I. R. Pala , M. Machler , M. F. Burz , J. W. Long , D. R. Rolison , Science 2017, 356, 415. 28450638 10.1126/science.aak9991

[21] D. Chao , W. Zhou , F. Xie , C. Ye , H. Li , M. Jaroniec , S.‐Z. Qiao , Sci. Adv. 2020, 6, eaba4098. 32494749 10.1126/sciadv.aba4098 PMC7244306

[22] Z. Pan , J. Yang , J. Jiang , Y. Qiu , J. Wang , Mater. Today Energy 2020, 18, 100523.

[23] Y. Zhang , Y. Wu , W. You , M. Tian , P.‐W. Huang , Y. Zhang , Z. Sun , Y. Ma , T. Hao , N. Liu , Nano Lett. 2020, 20, 4700. 32453958 10.1021/acs.nanolett.0c01776

[24] J. Liu , J. Long , Z. Shen , X. Jin , T. Han , T. Si , H. Zhang , Adv. Sci. 2021, 8, 2004689. 10.1002/advs.202004689 PMC8061350 33898202

[25] Y. Tian , Y. An , C. Wei , B. Xi , S. Xiong , J. Feng , Y. Qian , ACS Nano 2019, 13, 11676. 31585034 10.1021/acsnano.9b05599

[26] H. Tan , D. Chen , W. Liu , C. Liu , B. Lu , X. Rui , Q. Yan , Batteries Supercaps 2020, 3, 254.

[27] L. Dong , W. Yang , W. Yang , H. Tian , Y. Huang , X. Wang , C. Xu , C. Wang , F. Kang , G. Wang , Chem. Eng. J. 2020, 384, 123355.

[28] X. Wang , S. Zheng , F. Zhou , J. Qin , X. Shi , S. Wang , C. Sun , X. Bao , Z.‐S. Wu , Natl. Sci. Rev. 2020, 7, 64. 34692018 10.1093/nsr/nwz070 PMC8288951

[29] K. Jiang , Z. Zhou , X. Wen , Q. Weng , Small 2021, 17, 2007389. 10.1002/smll.202007389 33656244

[30] W. Xu , C. Liu , Q. Wu , W. Xie , W.‐Y. Kim , S.‐Y. Lee , J. Gwon , J. Mater. Chem. A 2020, 8, 18327.

[31] F. Mo , Z. Chen , G. Liang , D. Wang , Y. Zhao , H. Li , B. Dong , C. Zhi , Adv. Energy Mater. 2020, 10, 2000035.

[32] S. J. Joshi , R. M. M. Abed , Environ. Processes 2017, 4, 463.

[33] C. H. Park , Y. K. Kang , S. S. Im , J. Appl. Polym. Sci. 2004, 94, 248.

[34] J. Sheng , R. Wang , R. Yang , Materials 2019, 12, 2.

[35] N. Sun , F. Lu , Y. Yu , L. Su , X. Gao , L. Zheng , ACS Appl. Mater. Interfaces 2020, 12, 11778. 32073813 10.1021/acsami.0c00325

[36] H. Miao , B. Chen , S. Li , X. Wu , Q. Wang , C. Zhang , Z. Sun , H. Li , J. Power Sources 2020, 450, 227653.

[37] L. Yin , J. Scharf , J. Ma , J.‐M. Doux , C. Redquest , V. L. Le , Y. Yin , J. Ortega , X. Wei , J. Wang , Y. S. Meng , Joule 2021, 5, 228.

[38] P. Yang , C. Feng , Y. Liu , T. Cheng , X. Yang , H. Liu , K. Liu , H. J. Fan , Adv. Energy Mater. 2020, 10, 2002898.

[39] Y. Gogotsi , P. Simon , Science 2011, 334, 917. 22096182 10.1126/science.1213003

[40] J. Liu , C. Guan , C. Zhou , Z. Fan , Q. Ke , G. Zhang , C. Liu , J. Wang , Adv. Mater. 2016, 28, 8732. 27562134 10.1002/adma.201603038

[41] J. Liu , N. Nie , J. Wang , M. Hu , J. Zhang , M. Li , Y. Huang , Mater. Today Energy 2020, 16, 100372.

[42] W. Sun , F. Wang , S. Hou , C. Yang , X. Fan , Z. Ma , T. Gao , F. Han , R. Hu , M. Zhu , C. Wang , J. Am. Chem. Soc. 2017, 139, 9775. 28704997 10.1021/jacs.7b04471

[43] Y. Huang , W. S. Ip , Y. Y. Lau , J. Sun , J. Zeng , N. S. S. Yeung , W. S. Ng , H. Li , Z. Pei , Q. Xue , Y. Wang , J. Yu , H. Hu , C. Zhi , ACS Nano 2017, 11, 8953. 28813141 10.1021/acsnano.7b03322

[44] T. Zhao , G. Zhang , F. Zhou , S. Zhang , C. Deng , Small 2018, 14, 1802320. 10.1002/smll.201802320 30106506

[45] J. Liu , M. Chen , L. Zhang , J. Jiang , J. Yan , Y. Huang , J. Lin , H. J. Fan , Z. X. Shen , Nano Lett. 2014, 14, 7180. 25402965 10.1021/nl503852m

[46] L. Hu , H. Wu , F. L.a Mantia , Y. Yang , Y. Cui , ACS Nano 2010, 4, 5843. 20836501 10.1021/nn1018158

[47] B. Yao , L. Yuan , X. Xiao , J. Zhang , Y. Qi , J. Zhou , J. Zhou , B. Hu , W. Chen , Nano Energy 2013, 2, 1071.

[48] X. Xiao , T. Li , Z. Peng , H. Jin , Q. Zhong , Q. Hu , B. Yao , Q. Luo , C. Zhang , L. Gong , J. Chen , Y. Gogotsi , J. Zhou , Nano Energy 2014, 6, 1.

[49] Z. Song , T. Ma , R. Tang , Q. Cheng , X. Wang , D. Krishnaraju , R. Panat , C. K. Chan , H. Yu , H. Jiang , Nat. Commun. 2014, 5, 3140. 24469233 10.1038/ncomms4140

[50] S. Berchmans , A. J. Bandodkar , W. Jia , J. Ramírez , Y. S. Meng , J. Wang , J. Mater. Chem. A 2014, 2, 15788.

[51] R. Guo , J. Chen , B. Yang , L. Liu , L. Su , B. Shen , X. Yan , Adv. Funct. Mater. 2017, 27, 1702394.

[52] B. Nagar , D. P. Dubal , L. Pires , A. Merkoçi , P. Gómez‐Romero , ChemSusChem 2018, 11, 1849. 29786963 10.1002/cssc.201800426

[53] M. J. Gonzalez‐Guerrero , F. A. Gomez , Sens. Actuators, B 2018, 273, 101.

[54] Y. Wang , H. Kwok , W. Pan , H. Zhang , D. Y. C. Leung , J. Power Sources 2019, 414, 278.

[55] L. Zeng , S. Chen , M. Liu , H.‐M. Cheng , L. Qiu , ACS Appl. Mater. Interfaces 2019, 11, 46776. 31755259 10.1021/acsami.9b15866

[56] Z. Lv , Y. Luo , Y. Tang , J. Wei , Z. Zhu , X. Zhou , W. Li , Y. Zeng , W. Zhang , Y. Zhang , D. Qi , S. Pan , X. J. Loh , X. Chen , Adv. Mater. 2018, 30, 1704531. 10.1002/adma.201704531 29134702

[57] F. Liu , L. Zeng , Y. Chen , R. Zhang , R. Yang , J. Pang , L. Ding , H. Liu , W. Zhou , Nano Energy 2019, 61, 18.

[58] M. Yao , Z. Yuan , S. Li , T. He , R. Wang , M. Yuan , Z. Niu , Adv. Mater. 2021, 33, 2008140. 10.1002/adma.202008140 33533121

[59] M. Wang , D. Vecchio , C. Wang , A. Emre , X. Xiao , Z. Jiang , P. Bogdan , Y. Huang , N. A. Kotov , Sci. Rob. 2020, 5, eaba1912. 10.1126/scirobotics.aba1912 33022630

[60] F. Brunetti , A. Operamolla , S. Castro‐Hermosa , G. Lucarelli , V. Manca , G. M. Farinola , T. M. Brown , Adv. Funct. Mater. 2019, 29, 1806798.

[61] L. Gao , L. Chao , M. Hou , J. Liang , Y. Chen , H.‐D. Yu , W. Huang , npj Flexible Electron. 2019, 3, 4.

[62] Q. Tang , H. Guo , P. Yan , C. Hu , EcoMat 2020, 2, 12060.

