# Supplementary material for: Printed Zinc Paper Batteries
Source: Adv Sci (Weinh). 2021 Nov 5;9(2):2103894. doi: 10.1002/advs.202103894 (PMC8760176; doi:10.1002/advs.202103894)
Supplement: Supplementary file 1 — Supporting Information [file ADVS-9-2103894-s001.pdf]

© 2021 Wiley-VCH GmbH

ADVANCED SCIENCE
Open Access

Supporting Information

for *Adv. Sci.*, DOI: 10.1002/advs.202103894

Printed Zinc Paper Batteries

Peihua Yang, Jia Li, Seok Woo Lee, and Hong Jin Fan**

((Supporting Information can be included here using this template))

Supporting Information

Printed Zinc Paper Batteries

Peihua Yang, Jia Li, Seok Woo Lee, and Hong Jin Fan**

1. Experimental details

Materials: Acrylamide (AR, 99%) and *N*,*N*'-methylenebis(acrylamide) (AR) were purchased from Macklin. $(CH_3COO)_2Mn•4H_2O$ (99%), $KMnO_4$ (99%), $ZnCl_2$ (98%), $ZnSO_4•7H_2O$ (99%), $MnSO_4•H_2O$ (99%), $NiCl_2•6H_2O$ (98%), KOH (≥85%), LiOH (98%), $K_2S_2O_8$ (99%), *N*-methyl-2-pyrrolidone (NMP, 99%), polyvinylidene difluoride (PVDF, average Mw ~534,000, powder) and zinc dust (98%) were purchased from Sigma Aldrich. Carbon black was purchased from Alpha Aesar (Super P, H30253). Cellulose filter paper (Whatman) was used for hydrogel reinforced paper fabrication. Gold foil was purchased from Yongbo Gold Foil (thickness of ~120 nm, Nanjing, China; ~100 \$ m^{-2}). All reagents were used without further purification.

Preparation of hydrogel reinforced cellulose paper (HCP): The hydrogel was synthesized directly in the cellulose fibers. Firstly, cellulose filter paper was immersed into 2 mol L^{-1} acrylamide solution containing 4×10^{-4} mol L^{-1} *N*,*N*'-methylenebis(acrylamide) as a crosslinking agent and 4×10^{-4} mol L^{-1} potassium persulfate as initiator. The wettish cellulose paper was then sandwiched between two pieces of polyethylene covered glasses and heated at 60 °C with nitrogen protection for 6 hours. The obtained HCP was vacuum dried, and excess hydrogel on the surface was removed by polishing with sandpapers.

MnO_2 synthesis: The MnO_2 was synthesized by a co-precipitation method. Firstly, 100 mL 0.12 mol L^{-1} $(CH_3COO)_2Mn$ was added dropwise into 100 mL aqueous solution containing 0.08 mol L^{-1} $KMnO_4$. The mixed solution was stirred for 1 hours. The obtained dark brown precipitate was centrifuged and washed several times using deionized water and annealed at 450 °C for 2 hours to finally obtain the MnO_2 product.

$Ni(OH)_2$ synthesis: The $Ni(OH)_2$ was synthesized by a co-precipitation method. Firstly, 100 mL 0.5 mol L^{-1} $NiCl_2$ was added dropwise into 100 mL aqueous solution containing 1 mol L^{-1} KOH with nitrogen protection. The mixed solution was stirred for 1 hours. The obtained green precipitate was centrifuged and washed several times using deionized water and dried at 40 °C in a vacuum oven to finally obtain the $Ni(OH)_2$ product.

Paper battery fabrication: The Zn anode ink was formulated by mixing Zn dust, ZnO, carbon black and PVDF in a 1:0.2:0.05:0.05 weight ratio with NMP solvent through a conditioning mixer (THINKYMIXER, AR-100). Similarly, the $Ni(OH)_2$ ink (or MnO_2 ink) was formulated by mixing $Ni(OH)_2$ (or MnO_2), carbon black and PVDF in a 1:0.2:0.2 weight ratio. The anode

and cathode inks were printed on each side of dry HCP by screen printing and dried under vacuum at 40 °C. The effective mass loading of Zn, $Ni(OH)_2$ and MnO_2 is about 15, 6 and 6 mg cm^{-2}, respectively. For Ni-Zn battery, the printed battery was immersed in a mixed electrolyte of 6 M KOH and 1 M LiOH saturated with zinc powers. For the Mn-Zn battery, a hybrid electrolyte containing of 4 M $ZnCl_2$, 0.5 M $ZnSO_4$ and 0.5 M $MnSO_4$ was used. Gold foil was employed as current collectors for both electrodes.

Characterizations: Morphological analyses were performed on a scanning electron microscope (SEM, JEOL FESEM 7600F) equipped with an energy-dispersive X-ray spectroscopy (EDS) detector. Optical microscope images were taken by Nikon Eclipse Ci with a CCD camera. X-ray diffraction (XRD) was operated on a Bruker XRD analyzer (D8 ADVANCE). Electrochemical measurements were proceeded on Zahner Zennium and Biologic (VMP3) electrochemical workstations. The battery impedance was potentiostatically measured at open circuit voltage after charging with an AC oscillation of 10 mV amplitude over the frequency range from100 kHz to 100 mHz. The battery cycling stability was performed on Neware battery cycler (CT-4008-5V10mA-164, Shenzhen, China). Bending test was performed on a home-made platform based on LEGO Mindstorms EV3 set. Photo-charging of the paper battery was conducted by using a LED solar simulator (Newport LSH-7320 ABA). The thermogravimetric analysis of liquid electrolytes used for hydrogel swelling was performed via a thermal analyzer (Shimadzu DTG-60H TG/DTA). The strain-stress tests were conducted on a tensile tester (Shimadzu, EZ Test, EZ-SX) at a speed of 0.1 mm s^{-1}. The biodegradation tests were conducted in a roof garden with natural soil at NTU campus in Singapore.

Calculations: The capacity of the battery was calculated by $C = I\Delta t/A$, where I is the discharge current, Δt is the discharge time, and A is the area of paper battery. The energy density (E) and power density (P) of the battery was calculated by $E = \int_0^{\Delta t} IU(t)\mathrm{d}t\,/V$ and $P = E/\Delta t$, respectively, where $U(t)$ is the discharge voltage at t, dt is time differential, and V is the entire device volume. The vapor pressure of the electrolytes was determined by using thermogravimetry method, as described in our previous study.[1]

2. Supplementary Figures

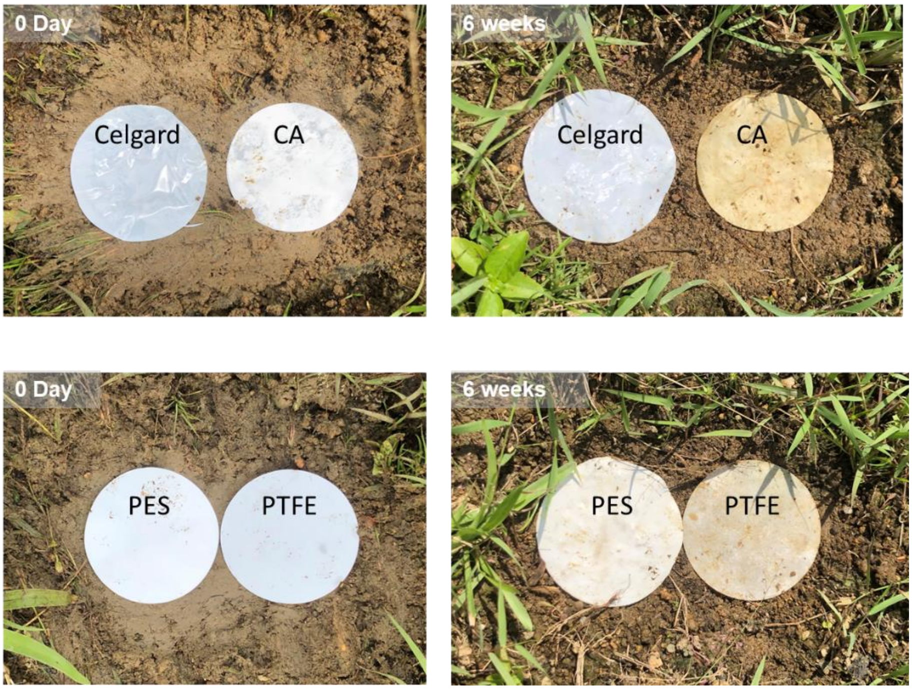

Figure S1. Biodegradation test of Celgard 2325, CA, PES and PTFE films (four samples with the same diameter of 47 mm).

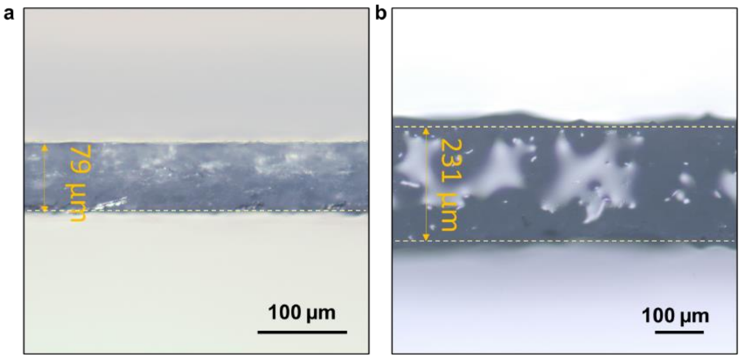

Figure S2. Cross-section optical microscope images of (a) dry HCP and (b) HCP after swelling in electrolyte. Thickness of the film were measured and labeled.

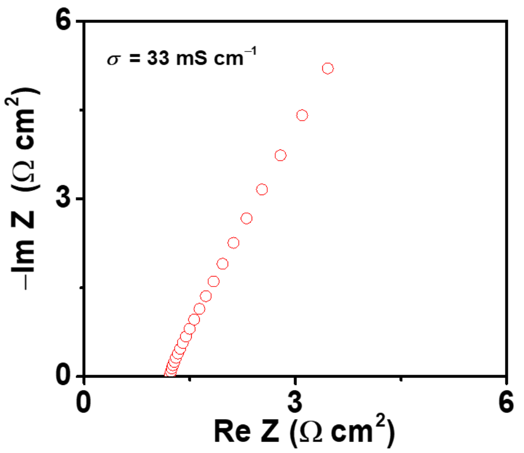

Figure S3. Impedance of HCP, which was swollen in 6 mol L^{-1} KOH/1 mol L^{-1} LiOH mixture electrolyte.

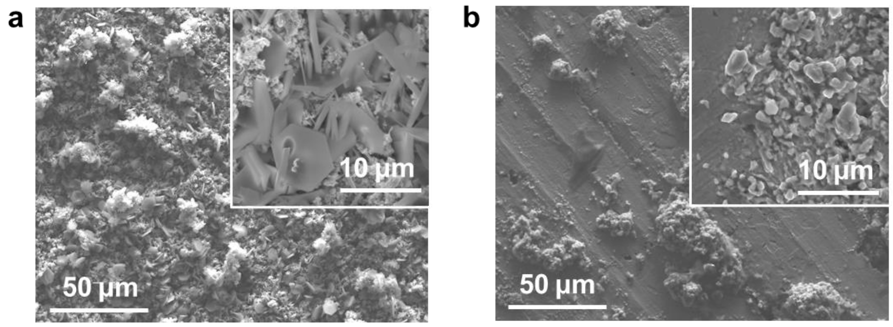

Figure S4. SEM images of zinc foil after galvanostatic plating/stripping cycling with (a) liquid electrolyte and (b) HCP electrolyte at a current density of 5 mA cm^{-2} and a cut-off capacity of 1 mAh cm^{-2}.

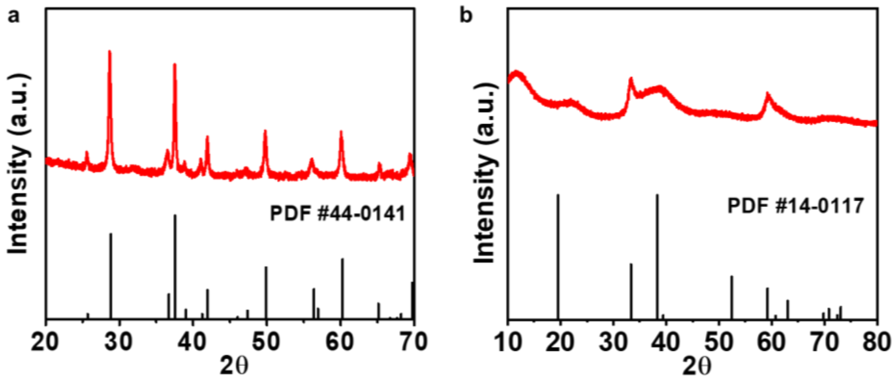

Figure S5. XRD patterns of synthesized cathode materials (a) MnO_2 and (b) $Ni(OH)_2$. The MnO_2 powers are generally crystalline. The $Ni(OH)_2$ powers are mostly amorphous due to the room temperature synthesis.[2] Characteristic peaks are observed at 33.1° (100), 38.5° (101), and 59.1° (110).

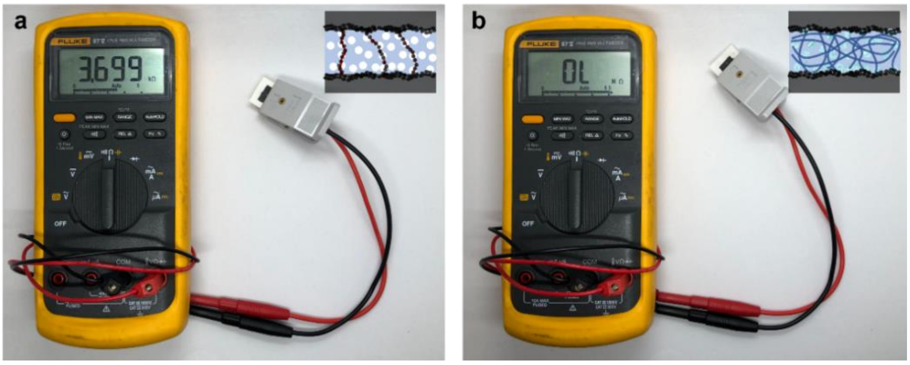

Figure S6. Short-circuit investigation when electrodes are printed on both side of (a) CP and (b) HCP. The printed materials can penetrate the porous CP and cause a short circuit (the dry device deliver an internal resistance of ~3.7 kΩ), while the hydrogel filled in HCP provides a dense separator and effectively prevents the mixing of inks, thus avoids short of a battery (see schematics in the insets).

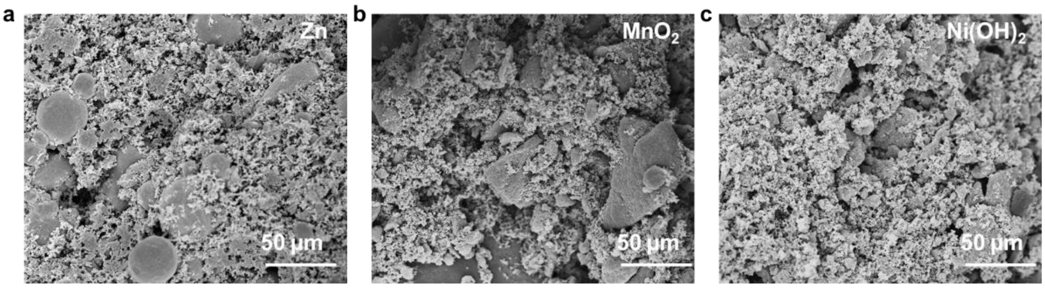

Figure S7. SEM images of printed (a) Zn, (b) MnO_2 and (c) $Ni(OH)_2$ electrodes.

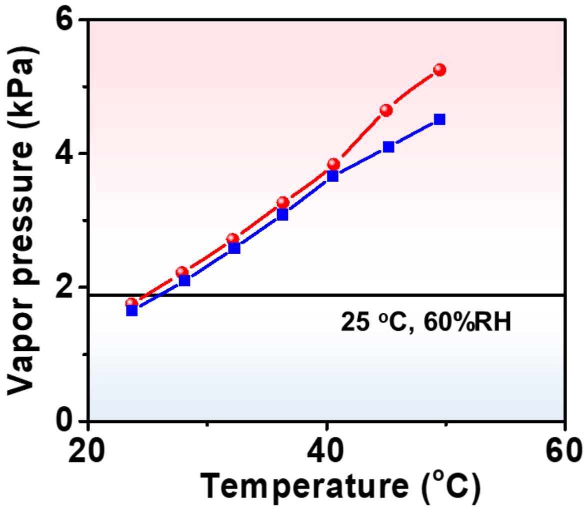

Figure S8. Vapor pressure of the electrolyte changes with temperature, blue square: 4 mol L^{-1} $ZnCl_2$/0.5 mol L^{-1} $ZnSO_4$/0.5 mol L^{-1} $MnSO_4$; red sphere: 6 mol L^{-1} KOH/1 mol L^{-1} LiOH. Black line indicates the partial pressure of water at 25 °C and 60% relative humidity ambient environment.

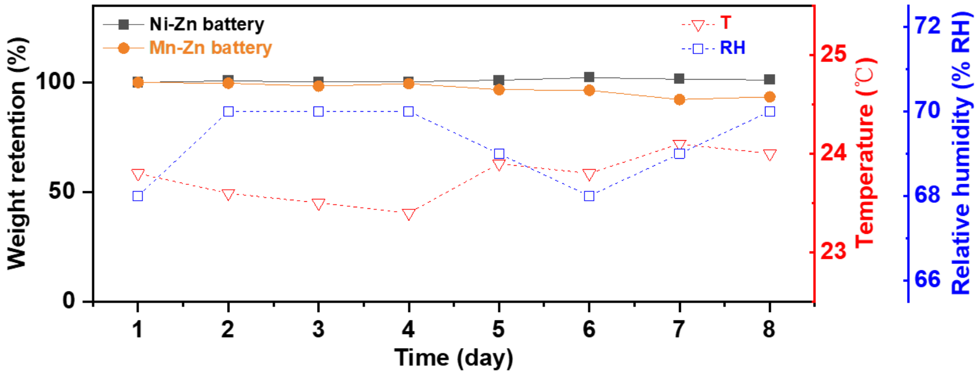

Figure S9. Weight change of the printed batteries in ambient lab condition without packaging.

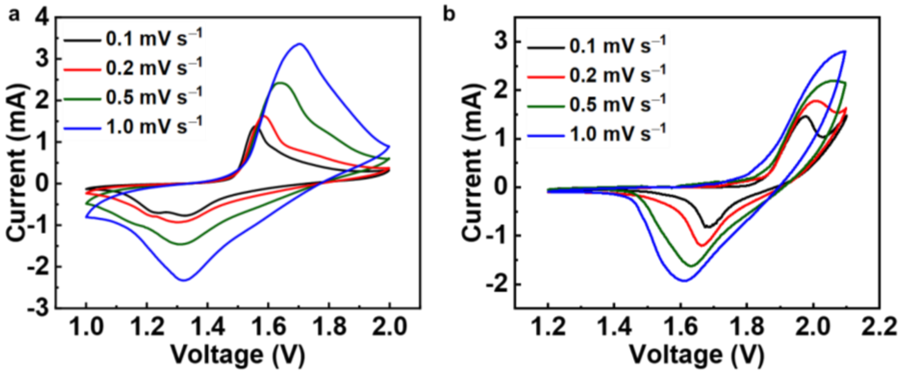

Figure S10. The cyclic voltammetry curves of (a) Mn-Zn and (b) Ni-Zn batteries at various scan rates.

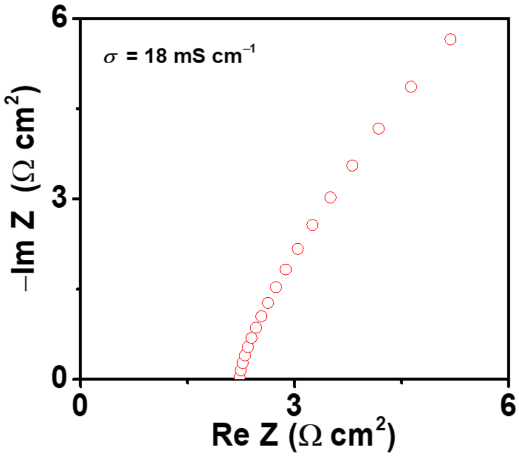

Figure S11. Impedance of HCP, which was swollen in 4 mol L^{-1} $ZnCl_2$/0.5 mol L^{-1} $ZnSO_4$/0.5 mol L^{-1} $MnSO_4$ mixture electrolyte.

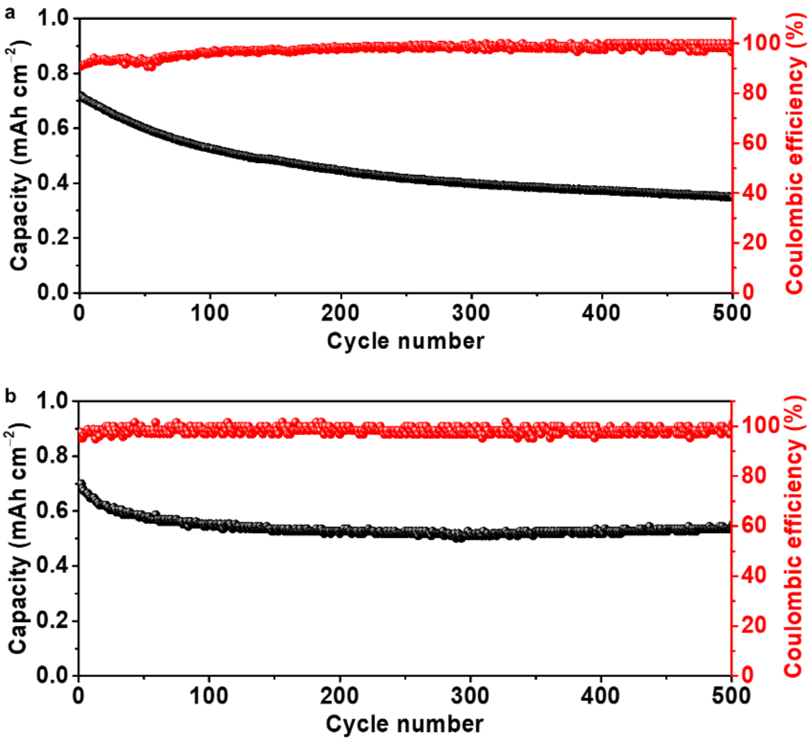

Figure S12. Cycling stability test of (a) Ni-Zn and (b) Mn-Zn batteries at a current density of 2 mA cm^{-2}.

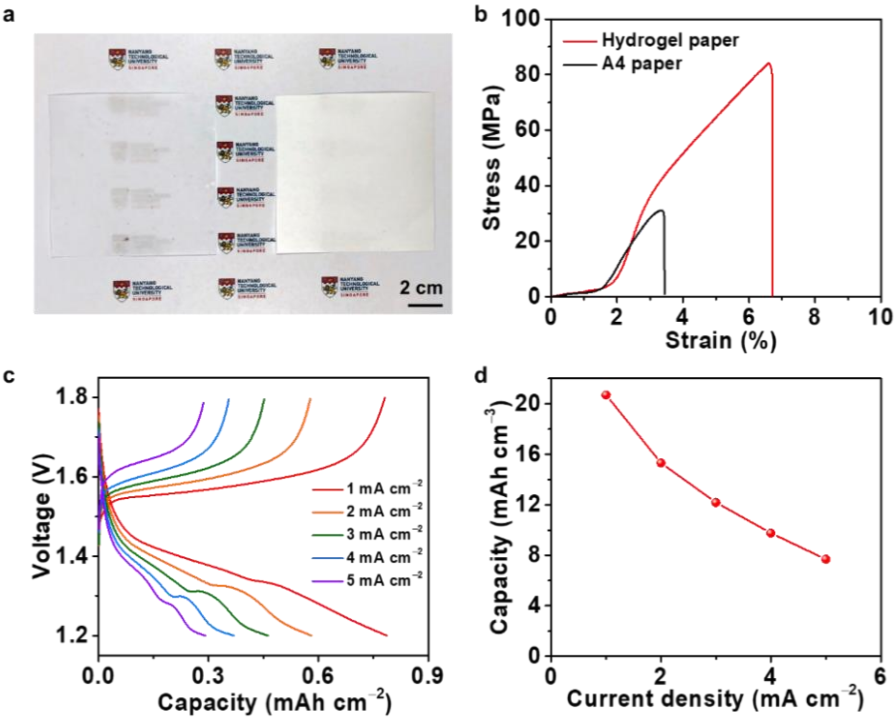

Figure S13. Commercial copy paper trial to realize hydrogel paper and printable battery. (a) Optical image of hydrogel paper (left) and original copy paper (right). (b) Stress-strain curves. (c) The charge and discharge curves of printed Mn-Zn batteries based on the produced hydrogel paper. (d) Volumetric capacity calculated from discharge curves.

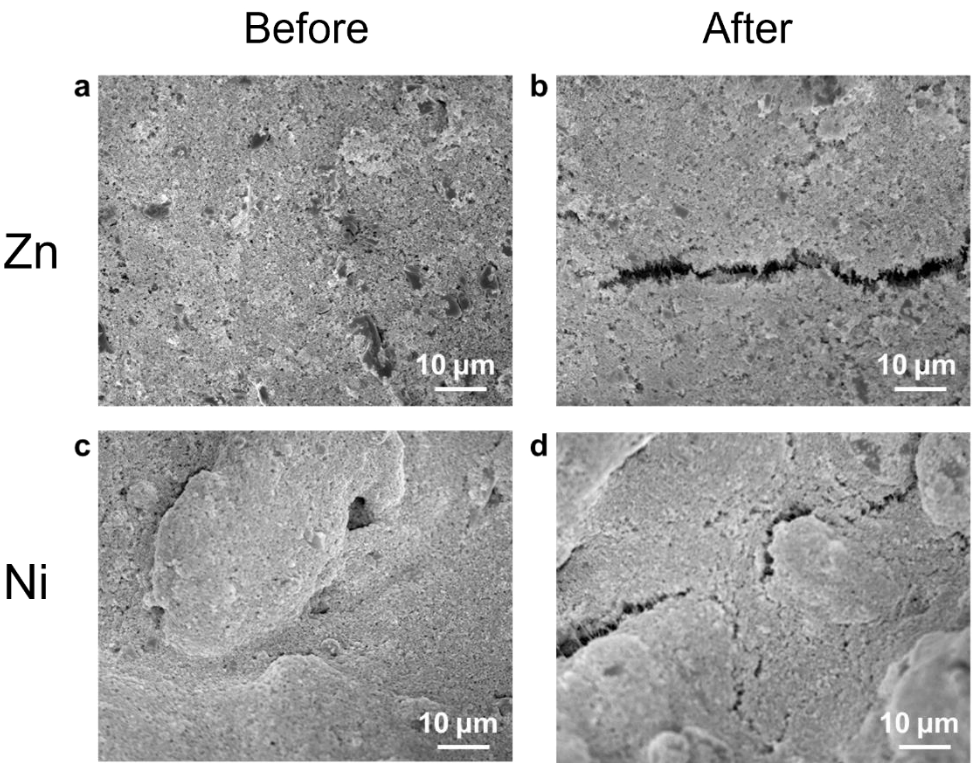

Figure S14. SEM images of the Zn anode (a) before and (b) after bending 1000 times, Ni cathode (c) before and (d) after bending 1000 times.

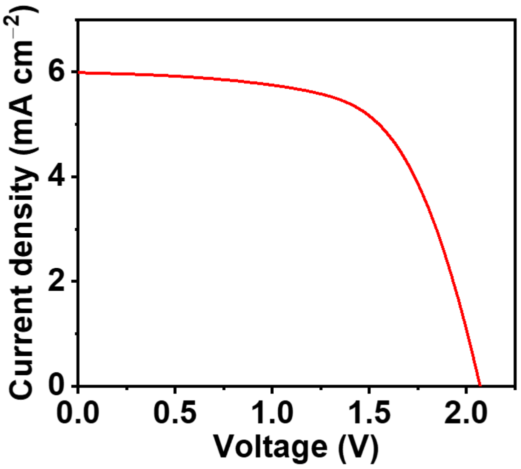

Figure S15. Photocurrent density versus voltage of the commercial solar cell under standard simulated sunlight.

3. Supplementary Videos

Vedio S1. Mechanical deformation during battery operation.

Vedio S2. Cuttability demonstration of paper battery.

4. Supplementary Table

Table S1. Overview of paper-based energy storage devices.

**Device[a]**	**Materials[b]**	**Device configuration**	**Thickness of device (mm)**	**Working voltage (V)**	**Capacity[c] (mAh cm^{-3})**	**Energy density (mWh cm^{-3})**	**Year & Reference**
LIB	CNT, LTO, LCO	Sandwich	0.3	2.7	19.5	-	2010[3]
SC	polyaniline	Sandwich	0.22	0.8	-	0.32	2013[4]
SC	CNT	Sandwich	0.13	~0.8	-	1.5	2014[5]
LIB	LTO, LCO	Sandwich	0.38	2.0-2.5	5.3	-	2014[6]
Ag-Zn battery	Ag, Zn	Interdigitated	2	2	1.1	-	2014[7]
SC	Polypyrrole, MnO_2	Interdigitated	0.23	1.5	-	0.04	2017[8]
SC	graphene	Sandwich	0.7	0.8	-	0.03	2018[9]
Al-Ag battery	Al, Ag	Sandwich	1.2	1.5	0.9	-	2018[10]
Al-air battery	Al, MnO_2	Sandwich	0.48	1.2	15.7	-	2019[11]
LIB	LTO, LFP	Sandwich	~0.2	1.8	10.7	-	2019[12]
ZIB	MnO_2, Zn	Interdigitated	~0.2	1.8	~0.8	~0.9	2020[13]
**ZIB**	**MnO_2, Zn**	**Sandwich**	**~0.4**	**1.8~1.9**	**27.5**	**25.4**	**2021**
**ZIB**	**$Ni(OH)_2$, Zn**	**Sandwich**	**~0.4**	**1.4~1.6**	**20.0**	**26.6**	**2021**

[a] LIB: lithium-ion batteriy; SC: supercapacitor; ZIB: zinc-ion battery.
[b] CNT: carbon nanotube; LTO: $Li_4Ti_5O_{12}$; LCO: $LiCoO_2$; LFP: $LiFePO_4$.
[c] The capacity and energy density are calculated by using the entire device volume.

5. Supplementary References

[1] P. Yang, C. Feng, Y. Liu, T. Cheng, X. Yang, H. Liu, K. Liu, H. J. Fan, *Adv. Energy Mater.* **2020**, *10*, 2002898.
[2] Z. Wang, X. Meng, K. Chen, S. Mitra, *Adv. Mater. Interfaces* **2018**, *5*, 1701036.
[3] L. Hu, H. Wu, F. La Mantia, Y. Yang, Y. Cui, *ACS Nano* **2010**, *4*, 5843.
[4] B. Yao, L. Yuan, X. Xiao, J. Zhang, Y. Qi, J. Zhou, J. Zhou, B. Hu, W. Chen, *Nano Energy* **2013**, *2*, 1071.
[5] X. Xiao, T. Li, Z. Peng, H. Jin, Q. Zhong, Q. Hu, B. Yao, Q. Luo, C. Zhang, L. Gong, J. Chen, Y. Gogotsi, J. Zhou, *Nano Energy* **2014**, *6*, 1.
[6] Z. Song, T. Ma, R. Tang, Q. Cheng, X. Wang, D. Krishnaraju, R. Panat, C. K. Chan, H. Yu, H. Jiang, *Nat. Commun.* **2014**, *5*, 3140.
[7] S. Berchmans, A. J. Bandodkar, W. Jia, J. Ramírez, Y. S. Meng, J. Wang, *J. Mater. Chem. A* **2014**, *2*, 15788.
[8] R. Guo, J. Chen, B. Yang, L. Liu, L. Su, B. Shen, X. Yan, *Adv. Funct. Mater.* **2017**, *27*, 1702394.
[9] B. Nagar, D. P. Dubal, L. Pires, A. Merkoçi, P. Gómez-Romero, *ChemSusChem* **2018**, *11*, 1849.
[10] M. J. Gonzalez-Guerrero, F. A. Gomez, *Sens. Actuators, B* **2018**, *273*, 101.
[11] Y. Wang, H. Kwok, W. Pan, H. Zhang, D. Y. C. Leung, *J. Power Sources* **2019**, *414*, 278.
[12] L. Zeng, S. Chen, M. Liu, H.-M. Cheng, L. Qiu, *ACS Appl. Mater. Interfaces* **2019**, *11*, 46776.
[13] X. Wang, S. Zheng, F. Zhou, J. Qin, X. Shi, S. Wang, C. Sun, X. Bao, Z.-S. Wu, *Natl. Sci. Rev.* **2020**, *7*, 64.
